# 4-(5-Amino-1*H*-1,2,4-triazol-3-yl)pyridinium chloride monohydrate

**DOI:** 10.1107/S1600536811002406

**Published:** 2011-01-22

**Authors:** Victor M. Chernyshev, Elena V. Tarasova, Anna V. Chernysheva, Victor B. Rybakov

**Affiliations:** a South-Russia State Technical University, 346428 Novocherkassk, Russian Federation; b Department of Chemistry, Moscow State University, 119992 Moscow, Russian Federation

## Abstract

In the cation of the title compound, C_7_H_8_N_5_^+^·Cl^−^·H_2_O, the mean planes of the pyridine and 1,2,4-triazole rings form a dihedral angle of 2.3 (1)°. The N atom of the amino group adopts a trigonal–pyramidal configuration. The N atom of the pyridine ring is protonated, forming a chloride salt. In the crystal, inter­molecular N—H⋯O, N—H⋯N, N—H⋯Cl and O—H⋯Cl hydrogen bonds link the cations, anions and water mol­ecules into layers parallel to the (1, 0, 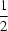) plane.

## Related literature

For the use of 3-pyridyl-substituted 5-amino-1,2,4-triazoles in the synthesis of biologically active compounds, see: Lipinski (1983 ▶); Ram (1988 ▶); Akahoshi *et al.* (1998 ▶); Young *et al.* (2001 ▶); Ouyang *et al.* (2005 ▶); Dolzhenko *et al.* (2007 ▶). For metal complexes of 3-pyridyl-substituted 5-amino-1,2,4-tri­azoles, see: Mishra *et al.* (1989 ▶); Ferrer *et al.* (2004 ▶); Castineiras & Garcia-Santos (2008 ▶). For a theoretical investigation of the protonation of *C*-amino-1,2,4-triazoles, see: Anders *et al.* (1997 ▶). For the crystal structures of protonated *C*-amino-1,2,4-triazoles, see: Lynch *et al.* (1999 ▶); Baouab *et al.* (2000 ▶); Bichay *et al.* (2006 ▶); Guerfel *et al.* (2007 ▶); Matulková *et al.* (2007 ▶). For the ionization constants (p*K*_α_) of 3-substituted 5-amino-1*H*-1,2,4-triazoles, see: Voronkov *et al.* (1976 ▶). For the ^1^H and ^13^C NMR spectra of 3-pyridyl-substituted 5-amino-1,2,4-triazoles, see: Dolzhenko *et al.* (2009*a* ▶). For typical NMR chemical shifts of 3-substituted 5-amino-1,2,4-triazoles and their salts, see: Chernyshev *et al.* (2010 ▶). For the crystal structures of 3-substituted 5-amino-1*H*-1,2,4-triazoles, see: Rusinov *et al.* (1991 ▶); Daro *et al.* (2000 ▶); Boechat *et al.* (2004 ▶); Dolzhenko *et al.* (2009*b* ▶,*c* ▶). For the crystal structures of 3(5)-pyridyl-substituted 1,2,4-triazoles protonated at the pyridine ring, see: Ren & Jian (2008 ▶); Xie *et al.* (2009 ▶); Du *et al.* (2009 ▶). For values of bond lengths in organic compounds, see: Allen *et al.* (1987 ▶). For the correlation of bond lengths with bond orders between *sp* ^2^-hybridized C and N atoms, see: Burke-Laing & Laing (1976 ▶).
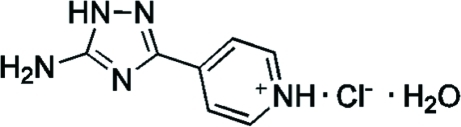


## Experimental

### 

#### Crystal data

C_7_H_8_N_5_^+^·Cl^−^·H_2_O
*M* *_r_* = 215.65
Monoclinic, 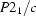

*a* = 5.3411 (5) Å
*b* = 24.656 (3) Å
*c* = 7.3488 (7) Å
β = 97.62 (2)°
*V* = 959.22 (18) Å^3^
*Z* = 4
Ag *K*α radiation
λ = 0.56085 Å
μ = 0.20 mm^−1^
*T* = 295 K
0.20 × 0.20 × 0.20 mm

#### Data collection

Enraf–Nonius CAD-4 diffractometer
Absorption correction: refined from Δ*F* (Walker & Stuart, 1983 ▶) *T*_min_ = 0.314, *T*_max_ = 0.961
2389 measured reflections
2389 independent reflections
1518 reflections with *I* > 2σ(*I*)
*R*_int_ = 0.000
1 standard reflections every 60 min  intensity decay: 2%

#### Refinement

*R*[*F* ^2^ > 2σ(*F* ^2^)] = 0.042
*wR*(*F* ^2^) = 0.109
*S* = 0.93
2389 reflections
151 parameters
H atoms treated by a mixture of independent and constrained refinement
Δρ_max_ = 0.26 e Å^−3^
Δρ_min_ = −0.24 e Å^−3^

### 

Data collection: *CAD-4 EXPRESS* (Enraf–Nonius, 1994 ▶); cell refinement: *CAD-4 EXPRESS*; data reduction: *XCAD4* (Harms & Wocadlo, 1995 ▶); program(s) used to solve structure: *SHELXS97* (Sheldrick, 2008 ▶); program(s) used to refine structure: *SHELXL97* (Sheldrick, 2008 ▶); molecular graphics: *ORTEP-3* (Farrugia, 1997 ▶); software used to prepare material for publication: *WinGX* (Farrugia, 1999 ▶).

## Supplementary Material

Crystal structure: contains datablocks global, I. DOI: 10.1107/S1600536811002406/aa2001sup1.cif

Structure factors: contains datablocks I. DOI: 10.1107/S1600536811002406/aa2001Isup2.hkl

Additional supplementary materials:  crystallographic information; 3D view; checkCIF report

## Figures and Tables

**Table 1:** Hydrogen-bond geometry (Å, °)

*D*—H⋯*A*	*D*—H	H⋯*A*	*D*⋯*A*	*D*—H⋯*A*
N1—H1⋯O1^i^	0.876 (19)	1.97 (2)	2.842 (2)	175.0 (18)
N16—H16⋯Cl1	0.85 (2)	2.22 (2)	3.0574 (18)	169 (2)
N51—H51*A*⋯Cl1^ii^	0.82 (2)	2.52 (2)	3.3092 (18)	160.5 (19)
N51—H51*B*⋯N4^iii^	0.82 (2)	2.15 (2)	2.963 (2)	174 (2)
O1—H1*A*⋯Cl1^iv^	0.89 (3)	2.29 (3)	3.159 (2)	167 (3)
O1—H1*B*⋯Cl1^v^	0.88 (3)	2.32 (3)	3.187 (2)	168 (2)

Symmetry codes: (i) 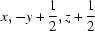; (ii) 
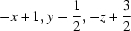
; (iii) 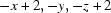; (iv) 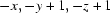; (v) 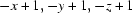.

**Fig. 1.**
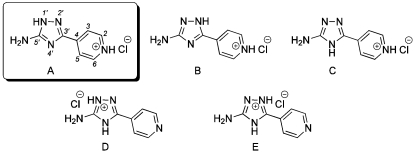
Possible tautomeric forms for hydrochloride of 5-amino-3-(pyridin-4-yl)-1,2,4-triazole.

**Fig. 2.**
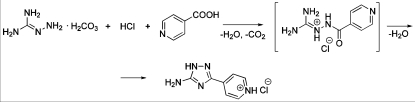
Synthesis of the title compound.

**Fig. 3.**
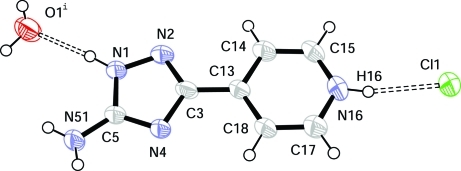
ORTEP-3 (Farrugia, 1997) plot of molecular structure of the title compound showing the atom-numbering scheme. Displacement ellipsoids are drawn at the 50% probability level. H atoms are presented as a small spheres of arbitrary radius.

**Fig. 4.**
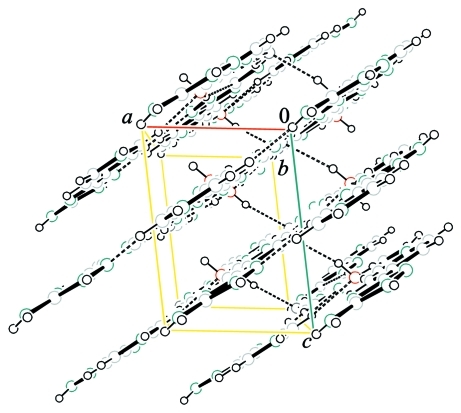
The molecular packing of the title compound along the b-axis showing molecular layers parallel to the plane (1, 0, 1/2). Hydrogen bonds are shown as dashed lines.

**Fig. 5.**
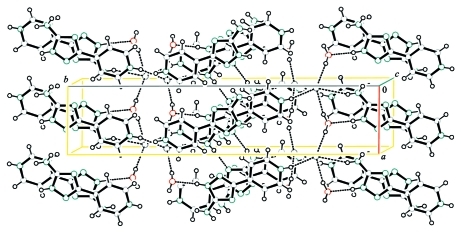
The molecular packing of the title compound along the c axis. Hydrogen bonds are shown as dashed lines.

## supplementary crystallographic information

### Comment

3-Pyridyl-substituted 5-amino-1,2,4-triazoles are used as reagents and ligands for the synthesis of biologically active compounds (Lipinski, 1983; Ram, 1988; Akahoshi *et al.*, 1998; Young *et al.*, 2001; Ouyang *et al.*, 2005; Dolzhenko *et al.*, 2007) and metal complexes (Mishra *et al.*, 1989; Ferrer *et al.*, 2004; Castineiras & Garcia-Santos, 2008). These substances are weak bases and form salts with acids. However, the positions of protonation centres of their molecules are open to question. For example, one would assume the existence of the tautomers A–E for the hydrochloride of 5-amino-3-(pyridin-4-yl)-1,2,4-triazole (Fig. 1). The tautomers A and D can be expected to be the most probable on the basis of the theoretical investigation of the protonation of *C*-amino-1,2,4-triazoles (Anders *et al.*, 1997) and X-ray studies of *C*-amino-1,2,4-triazolium salts (Lynch *et al.*, 1999; Baouab *et al.*, 2000; Bichay *et al.*, 2006; Guerfel *et al.*, 2007; Matulková *et al.*, 2007). Since knowledge of specific features of protonation of 3-pyridyl-substituted 5-amino-1,2,4-triazoles is essential for understanding their reactivity and biological properties, we investigated the structure of the hydrochloride of 5-amino-3-(pyridin-4-yl)-1,2,4-triazole in the solution and solid state. This compound was obtained by one-pot synthesis starting from aminoguanidine hydrogen carbonate, isonicotinic acid and hydrochloric acid (see Fig. 2 and Experimental).

By potentiometric titration with 0.1 *M* hydrochloric acid we established that the p*K*_α_ of the 5-amino-3-(pyridin-4-yl)-1,2,4-triazole in water is 4.68 (5) at 293 K. A model compound, 5-amino-3-phenyl-1*H*-1,2,4-triazole, which is protonated at the N^4^ of triazole cycle in acid solutions (Voronkov *et al.*, 1976), has the p*K*_α_ = 3.80 (3) at 293 K. Since the basicity of the 5-amino-3-(pyridin-4-yl)-1,2,4-triazole is almost eight times higher than the model compound, it is possible to assume that in water solution the pyridine rather than triazole cycle is protonated. In the ^13^C NMR spectrum of the hydrochloride of 5-amino-3-(pyridin-4-yl)-1,2,4-triazole in dimethyl sulfoxide (*DMSO*-*d*_6_), the signals of the triazole carbons C^3'^ and C^5'^ are observed at 153.71 and 157.83 ppm, correspondingly (for the chemical numbering scheme, see Fig. 1). These values are very close to the same signals of the unprotonated 5-amino-3-(pyridin-4-yl)-1H-1,2,4-triazole (Dolzhenko *et al.*, 2009*a*) and are typical for 5-amino-1*H*-1,2,4-triazoles (Chernyshev *et al.*, 2010). The chemical shift of the carbon connected to amino group is most representative. Thus, in the 5-amino- and 3-amino-4*H*-1,2,4-triazolium salts the signals of the same atoms are high field shifted to 149.3–154.7 ppm (Chernyshev *et al.*, 2010). Therefore, it could be concluded that the triazole cycle is unprotonated in DMSO solution of the studied salt. However, the signals of the carbons of the pyridine cycle of the hydrochloride (especially the carbons, connected with nitrogen atom) differ sufficiently from the ones of unprotonated 5-amino-3-(pyridin-4-yl)-1,2,4-triazole. In the unprotonated compound the signals of C^2^ and C^6^ are detected at 149.9 ppm (Dolzhenko *et al.*, 2009*a*), while in the hydrochloride they are observed at 142.9 ppm. Therefore, we can conclude that the pyridine cycle is protonated and the tautomeric form A is predominant in DMSO (Fig. 1). For unambiguous confirmation of the proposed structure, we performed an X-ray investigation of the title compound. In the ensuing discussion of the structure, the crystallographic numbering system will be used (Fig. 3). In accordance with the X-ray diffraction data, the studied compound in the crystal exists as the tautomer A (Fig. 3). The pyridine and triazole rings are almost coplanar, the dihedral angle between the planes of the rings is 2.3 (1)°. Bond lengths and angles in the triazole cycle are within the normal ranges and are comparable with those found in the other 3-substituted 5-amino-1*H*-1,2,4-triazoles (Rusinov *et al.*, 1991; Daro *et al.*, 2000; Boechat *et al.*, 2004; Dolzhenko *et al.*, 2009*b*,*c*). The nitrogen atom of the amino group is in a trigonal pyramidal configuration (sum of valence angles is 356.0° and deviates from the triazole plane by only 0.020 (3)Å. Conjugation between the unshared electron pair of N21 and the π-system of the triazole fragment leads to a shortening of the N21—C2 bond (1.330 (2)Å) relative to the standard length of a purely single Nsp^2^—Csp^2^ bond (1.43–1.45Å) (Burke-Laing & Laing, 1976; Allen *et al.*, 1987). Bond lengths and angles in the pyridine cycle are analogous to the ones in the pyridyl-substituted 1,2,4-triazoles, protonated at the pyridine cycle (Ren & Jian, 2008; Xie *et al.*, 2009; Du *et al.*, 2009).

The wide system of hydrogen bonds are found in crystal structure of title compound. Firstly, atom Cl1 form contact 2.22 (2)Å (Table 1) with atom H16 of pyridyl moiety; secondly, atom H3 of triazole moiety, forms contact with O1^i^ atom from water molecule (symmetry code: (i) *x*, -*y*+1/2, *z*+1/2; thirdly, atom H1B^i^ from water molecule forms contact with Cl1^ii^ (symmetry code: (ii) -*x*+1, *y*-1/2, -*z*+3/2). Thus, we can see chain of hydrogen bonds along [0 1 0]. These chains form layers parallel (1, 0, 1/2) plane (Fig. 4). These layers connected by hydrogen bonds with involving H1A atoms of water molecule (Fig. 5).

In the future, it would be interesting to investigate the structure of isomers of the studied compound, i.e. salts of the 5-amino-3-(pyridin-2-yl)-1,2,4-triazole and 5-amino-3-(pyridin-3-yl)-1,2,4-triazole in order to estimate the influence of structural peculiarities on protonation.

### Experimental

The title compound was prepared by the following procedure. A mixture of aminoguanidine hydrogen carbonate (5.53 g, 40.6 mmol), isonicotinic acid (5.01 g, 40.7 mmol) and 33.5% hydrochloric acid (5.0 ml) was heated to reflux for 15 min, then water was distilled off until the temperature of the reaction mixture raised to 448–453 K. The reaction mixture was heated at the same temperature for 6 h, cooled to ~373 K and dissolved in water (5 ml). The resulted solution was cooled to 276–278 K, the precipitate formed was isolated by filtration, recrystallized from 50% ethanol and dried at 403 K to give 6.82 g (85% yield) of yellowish powder, m. p. 579–581 K. Spectrum ^1^H NMR (600 MHz), δ: 6.61 (br s, 2H, NH_2_), 8.26 (d, J = 6.7 Hz, 2H, *Ar*), 8.85 (d, J = 6.7 Hz, 2H, *Ar*). Spectrum ^13^C NMR (150 MHz), δ: 121.78 (C^3^ and C^5^ of pyridine), 142.92 (C^2^ and C^6^ of pyridine), 145.51 (C^4^ of pyridine), 153.71 (C^3'^ of triazole), 157.83 (C^5'^ of triazole). MS (EI, 70 eV), m/*z* (%): 162 (10) [C_7_H_8_N_5_^+^], 161 (100) [C_7_H_7_N_5_^+^], 119 (26), 105 (45), 78 (46), 57 (68), 51 (71), 50 (38), 43 (38). Anal. Calcd for C_7_H_8_ClN_5_: C, 42.54; H, 4.08; N, 35.44. Found: C 42.35; H 4.19; N 35.18. The crystals of title compound suitable for *X*-ray analysis were grown by slow evaporation from water at room temperature.

### Refinement

C-bound H atoms were placed in calculated positions C—H 0.93Å and refined as riding, with *U*_iso_(H) = 1.2*U*_eq_(C). H-atoms forming hydrogen (N- and O-bound H atoms) bonds were found from difference Fourier map and refined independently. The initial experimental data were obtained for independent area of reciprocal space, but at the final stage of refinement procedure 'MERG 2' instruction was used and '*DIFABS* CAD4' (Walker & Stuart, 1983) was applied. As a result, we have FVAR = 1, *R*_int_ = 0.

### Crystal data

**Table:**

C_7_H_8_N_5_^+^·Cl^−^·H_2_O	*F*(000) = 448
*M**_r_* = 215.65	*D*_x_ = 1.493 Mg m^−^^3^
Monoclinic, *P*2_1_/*c*	Melting point = 579–581 K
Hall symbol: -P 2ybc	Ag *K*α radiation, λ = 0.56085 Å
*a* = 5.3411 (5) Å	Cell parameters from 25 reflections
*b* = 24.656 (3) Å	θ = 12.1–14.0°
*c* = 7.3488 (7) Å	µ = 0.20 mm^−^^1^
β = 97.62 (2)°	*T* = 295 K
*V* = 959.22 (18) Å^3^	Prism, yellow
*Z* = 4	0.20 × 0.20 × 0.20 mm

### Data collection

**Table:**

Enraf–Nonius CAD-4 diffractometer	1518 reflections with *I* > 2σ(*I*)
Radiation source: fine-focus sealed tube	*R*_int_ = 0.000
graphite	θ_max_ = 22.0°, θ_min_ = 1.3°
Non–profiled ω scans	*h* = −7→7
Absorption correction: part of the refinement model (Δ*F*) (Walker & Stuart, 1983)	*k* = 0→32
*T*_min_ = 0.314, *T*_max_ = 0.961	*l* = 0→9
2389 measured reflections	1 standard reflections every 60 min
2389 independent reflections	intensity decay: 2%

### Refinement

**Table:**

Refinement on *F*^2^	Primary atom site location: structure-invariant direct methods
Least-squares matrix: full	Secondary atom site location: difference Fourier map
*R*[*F*^2^ > 2σ(*F*^2^)] = 0.042	Hydrogen site location: inferred from neighbouring sites
*wR*(*F*^2^) = 0.109	H atoms treated by a mixture of independent and constrained refinement
*S* = 0.93	*w* = 1/[σ^2^(*F*_o_^2^) + (0.0642*P*)^2^] where *P* = (*F*_o_^2^ + 2*F*_c_^2^)/3
2389 reflections	(Δ/σ)_max_ = 0.001
151 parameters	Δρ_max_ = 0.26 e Å^−^^3^
0 restraints	Δρ_min_ = −0.24 e Å^−^^3^

### Special details

**Table:**

Geometry. All s.u.'s (except the s.u. in the dihedral angle between two l.s. planes) are estimated using the full covariance matrix. The cell s.u.'s are taken into account individually in the estimation of s.u.'s in distances, angles and torsion angles; correlations between s.u.'s in cell parameters are only used when they are defined by crystal symmetry. An approximate (isotropic) treatment of cell s.u.'s is used for estimating s.u.'s involving l.s. planes.
Refinement. Refinement of F^2^ against ALL reflections. The weighted R-factor wR and goodness of fit S are based on F^2^, conventional R-factors R are based on F, with F set to zero for negative F^2^. The threshold expression of F^2^ > 2σ(F^2^) is used only for calculating R-factors(gt) etc. and is not relevant to the choice of reflections for refinement. R-factors based on F^2^ are statistically about twice as large as those based on F, and R-factors based on ALL data will be even larger.

### Fractional atomic coordinates and isotropic or equivalent isotropic displacement parameters (Å^2^)

**Table:**

	*x*	*y*	*z*	*U*_iso_*/*U*_eq_	
Cl1	0.03502 (9)	0.29662 (2)	0.52890 (9)	0.0585 (2)	
N1	0.4858 (3)	−0.06015 (6)	0.7559 (2)	0.0421 (4)	
H1	0.458 (3)	−0.0952 (8)	0.751 (3)	0.038 (5)*	
N2	0.3363 (3)	−0.01915 (6)	0.6821 (2)	0.0438 (4)	
C3	0.4713 (3)	0.02346 (7)	0.7339 (3)	0.0380 (4)	
N4	0.7008 (3)	0.01345 (6)	0.8344 (2)	0.0401 (4)	
C5	0.7035 (3)	−0.03999 (7)	0.8458 (3)	0.0391 (4)	
C13	0.3868 (3)	0.07839 (7)	0.6860 (3)	0.0376 (4)	
C14	0.1558 (3)	0.08732 (8)	0.5770 (3)	0.0477 (5)	
H14	0.0536	0.0583	0.5337	0.057*	
C15	0.0833 (4)	0.13903 (8)	0.5355 (3)	0.0512 (5)	
H15	−0.0694	0.1455	0.4620	0.061*	
N16	0.2275 (3)	0.18070 (7)	0.5984 (3)	0.0514 (5)	
H16	0.197 (4)	0.2140 (9)	0.580 (3)	0.053 (6)*	
C17	0.4501 (4)	0.17327 (8)	0.7024 (3)	0.0535 (6)	
H17	0.5484	0.2031	0.7435	0.064*	
C18	0.5325 (3)	0.12272 (8)	0.7479 (3)	0.0468 (5)	
H18	0.6869	0.1177	0.8207	0.056*	
N51	0.8895 (3)	−0.07055 (7)	0.9294 (3)	0.0514 (5)	
H51A	0.869 (4)	−0.1036 (9)	0.934 (3)	0.048 (6)*	
H51B	0.994 (4)	−0.0532 (9)	0.998 (3)	0.063 (7)*	
O1	0.4229 (4)	0.67460 (7)	0.2570 (3)	0.0635 (5)	
H1A	0.309 (6)	0.6799 (12)	0.333 (4)	0.092 (11)*	
H1B	0.562 (6)	0.6872 (11)	0.319 (4)	0.092 (10)*	

### Atomic displacement parameters (Å^2^)

**Table:**

	*U*^11^	*U*^22^	*U*^33^	*U*^12^	*U*^13^	*U*^23^
Cl1	0.0424 (3)	0.0401 (3)	0.0893 (5)	0.0049 (2)	−0.0051 (3)	0.0026 (3)
N1	0.0292 (7)	0.0325 (8)	0.0603 (11)	−0.0045 (6)	−0.0100 (7)	0.0005 (8)
N2	0.0291 (7)	0.0428 (8)	0.0560 (10)	−0.0051 (6)	−0.0078 (7)	−0.0022 (8)
C3	0.0237 (8)	0.0429 (10)	0.0452 (11)	−0.0004 (7)	−0.0041 (7)	−0.0041 (8)
N4	0.0289 (7)	0.0330 (7)	0.0545 (10)	−0.0017 (6)	−0.0082 (7)	−0.0009 (7)
C5	0.0283 (8)	0.0359 (8)	0.0501 (11)	−0.0031 (7)	−0.0063 (7)	−0.0005 (8)
C13	0.0273 (8)	0.0392 (9)	0.0450 (10)	0.0019 (7)	−0.0002 (7)	−0.0004 (8)
C14	0.0320 (9)	0.0486 (11)	0.0584 (13)	−0.0007 (8)	−0.0092 (9)	−0.0014 (9)
C15	0.0312 (9)	0.0532 (12)	0.0650 (14)	0.0094 (8)	−0.0090 (9)	0.0057 (10)
N16	0.0413 (9)	0.0419 (9)	0.0686 (13)	0.0117 (7)	−0.0012 (8)	0.0048 (9)
C17	0.0393 (10)	0.0464 (11)	0.0706 (16)	0.0013 (9)	−0.0078 (10)	−0.0023 (10)
C18	0.0285 (9)	0.0426 (10)	0.0657 (13)	0.0029 (8)	−0.0076 (9)	0.0008 (10)
N51	0.0361 (8)	0.0326 (9)	0.0784 (14)	−0.0011 (7)	−0.0187 (8)	0.0002 (9)
O1	0.0426 (9)	0.0564 (10)	0.0867 (13)	0.0052 (7)	−0.0094 (10)	−0.0090 (9)

### Geometric parameters (Å, °)

**Table:**

N4—C5	1.320 (2)	C15—N16	1.330 (3)
N4—C3	1.367 (2)	C15—H15	0.9300
C5—N51	1.330 (2)	N16—C17	1.338 (3)
C5—N1	1.353 (2)	N16—H16	0.85 (2)
N1—N2	1.356 (2)	C17—C18	1.349 (3)
N1—H1	0.876 (19)	C17—H17	0.9300
N2—C3	1.302 (2)	C18—H18	0.9300
C3—C13	1.456 (2)	N51—H51A	0.82 (2)
C13—C18	1.383 (2)	N51—H51B	0.82 (2)
C13—C14	1.396 (2)	O1—H1A	0.89 (3)
C14—C15	1.355 (3)	O1—H1B	0.88 (3)
C14—H14	0.9300		
			
C5—N4—C3	102.47 (14)	N16—C15—C14	120.89 (17)
N4—C5—N51	126.56 (17)	N16—C15—H15	119.6
N4—C5—N1	109.53 (15)	C14—C15—H15	119.6
N51—C5—N1	123.90 (18)	C15—N16—C17	121.52 (18)
C5—N1—N2	110.08 (15)	C15—N16—H16	127.1 (16)
C5—N1—H1	120.6 (12)	C17—N16—H16	111.3 (16)
N2—N1—H1	129.3 (12)	N16—C17—C18	120.25 (18)
C3—N2—N1	102.18 (14)	N16—C17—H17	119.9
N2—C3—N4	115.73 (16)	C18—C17—H17	119.9
N2—C3—C13	122.53 (15)	C17—C18—C13	119.87 (17)
N4—C3—C13	121.73 (15)	C17—C18—H18	120.1
C18—C13—C14	118.66 (17)	C13—C18—H18	120.1
C18—C13—C3	120.84 (15)	C5—N51—H51A	118.8 (15)
C14—C13—C3	120.50 (16)	C5—N51—H51B	113.2 (17)
C15—C14—C13	118.80 (17)	H51A—N51—H51B	125 (2)
C15—C14—H14	120.6	H1A—O1—H1B	103 (3)
C13—C14—H14	120.6		
			
C3—N4—C5—N51	179.2 (2)	N2—C3—C13—C14	−1.6 (3)
C3—N4—C5—N1	0.1 (2)	N4—C3—C13—C14	177.42 (19)
N4—C5—N1—N2	0.4 (2)	C18—C13—C14—C15	0.2 (3)
N51—C5—N1—N2	−178.75 (19)	C3—C13—C14—C15	179.92 (19)
C5—N1—N2—C3	−0.8 (2)	C13—C14—C15—N16	−0.6 (3)
N1—N2—C3—N4	0.9 (2)	C14—C15—N16—C17	0.9 (3)
N1—N2—C3—C13	179.91 (17)	C15—N16—C17—C18	−0.7 (3)
C5—N4—C3—N2	−0.6 (2)	N16—C17—C18—C13	0.3 (3)
C5—N4—C3—C13	−179.68 (18)	C14—C13—C18—C17	0.0 (3)
N2—C3—C13—C18	178.20 (19)	C3—C13—C18—C17	−179.8 (2)
N4—C3—C13—C18	−2.8 (3)		

### Hydrogen-bond geometry (Å, °)

**Table:**

*D*—H···*A*	*D*—H	H···*A*	*D*···*A*	*D*—H···*A*
N1—H1···O1^i^	0.876 (19)	1.97 (2)	2.842 (2)	175.0 (18)
N16—H16···Cl1	0.85 (2)	2.22 (2)	3.0574 (18)	169 (2)
N51—H51A···Cl1^ii^	0.82 (2)	2.52 (2)	3.3092 (18)	160.5 (19)
N51—H51B···N4^iii^	0.82 (2)	2.15 (2)	2.963 (2)	174 (2)
O1—H1A···Cl1^iv^	0.89 (3)	2.29 (3)	3.159 (2)	167 (3)
O1—H1B···Cl1^v^	0.88 (3)	2.32 (3)	3.187 (2)	168 (2)

Symmetry codes: (i) *x*, −*y*+1/2, *z*+1/2; (ii) −*x*+1, *y*−1/2, −*z*+3/2; (iii) −*x*+2, −*y*, −*z*+2; (iv) −*x*, −*y*+1, −*z*+1; (v) −*x*+1, −*y*+1, −*z*+1.
